# Phylogenetic and comparative genomics of the family *Leptotrichiaceae* and introduction of a novel fingerprinting MLVA for *Streptobacillus moniliformis*

**DOI:** 10.1186/s12864-016-3206-0

**Published:** 2016-11-03

**Authors:** Tobias Eisenberg, Ahmad Fawzy, Werner Nicklas, Torsten Semmler, Christa Ewers

**Affiliations:** 1 Abteilung Veterinärmedizin, Landesbetrieb Hessisches Landeslabor (LHL), Schubertstr. 60/H13, D-35392 Giessen, Germany; 2 Department of Medicine and Infectious Diseases, Faculty of Veterinary Medicine, Cairo University, Giza Square, 12211 Egypt; 3 Deutsches Krebsforschungszentrum, D-69120 Heidelberg, Germany; 4 Robert Koch-Institut, D-13353 Berlin, Germany; 5 Institut für Hygiene und Infektionskrankheiten der Tiere, Justus-Liebig-Universität Giessen, D-35392 Giessen, Germany

**Keywords:** Next generation sequencing, Multi locus variable number tandem repeat analysis (MVLA), Phylogeny, Typing, Fingerprinting, *Streptobacillus*, *Leptotrichiaceae*

## Abstract

**Background:**

The *Leptotrichiaceae* are a family of fairly unnoticed bacteria containing both microbiota on mucous membranes as well as significant pathogens such as *Streptobacillus moniliformis*, the causative organism of streptobacillary rat bite fever. Comprehensive genomic studies in members of this family have so far not been carried out. We aimed to analyze 47 genomes from 20 different member species to illuminate phylogenetic aspects, as well as genomic and discriminatory properties.

**Results:**

Our data provide a novel and reliable basis of support for previously established phylogeny from this group and give a deeper insight into characteristics of genome structure and gene functions. Full genome analyses revealed that most *S. moniliformis* strains under study form a heterogeneous population without any significant clustering. Analysis of infra-species variability for this highly pathogenic rat bite fever organism led to the detection of three specific variable number tandem analysis loci with high discriminatory power.

**Conclusions:**

This highly useful and economical tool can be directly employed in clinical samples without laborious prior cultivation. Our and prospective case-specific data can now easily be compared by using a newly established MLVA database in order to gain a better insight into the epidemiology of this presumably under-reported zoonosis.

**Electronic supplementary material:**

The online version of this article (doi:10.1186/s12864-016-3206-0) contains supplementary material, which is available to authorized users.

## Background

The *Leptotrichiaceae* are a family of underexplored and rarely isolated microorganisms within the phylum Fusobacteria containing both species known from certain pathologies as well as colonising members of the resident microbiota. Many if not all species of the *Leptotrichiaceae* inhabit the oral cavities, gastrointestinal or urogenital tracts of humans and animals [1–3]. One of the reasons they are rarely encountered is the obligate anaerobic or capnophilic growth dependence of these fastidious bacteria and the usual presence of a high number of concomitant microorganisms. Some members of this family are well known pathogens, such as *Streptobacillus* (*S.*) *moniliformis*, one of the two causative organisms of the bacterial zoonosis rat bite fever [4]. Recently, a number of novel species have been described, most of which could be attributed to clinical disease [5–8]. It can also be concluded from numerous phylotypes, *Leptotrichiaceae* normally colonize mucous membranes [9–15], but when introduced into new tissue or host sites they are also able to shift their pathogenic potential and cause severe and even life-threatening disease. With increasing availability of next generation sequencing a number of single genomes have been published [6, 16–20]. However, almost no comprehensive genomic studies including these microorganisms have been completed, nor have virulence properties been identified in these species. Phylogenetic studies and identifications within the phylum Fusobacteria have been carried out and based on single or multiple gene sequences such as 16S rRNA, 16S–23S rRNA internal transcribed spacer, *gyrB*, *groEL*, *recA*, *rpoB*, conserved indels and genes for group-specific proteins, 43-kDa outer membrane protein and zinc protease [18, 21–30]. In an attempt to characterize different members of this phylum Gupta & Seti proposed various conserved signature indels (CSIs) in amino acid sequences for the *Leptotrichiaceae* from which three CSIs were found to be specific for this family [31]. On the other hand, no detailed phylogenetic and comparative genome studies dedicated to *Leptotrichiaceae* have been published up to now. Furthermore, and due to a general paucity of strains and attempts to differentiate members from the same species there is currently no tool available to type isolates in order to prove transmission chains. Our data, presented here, were derived from 46 complete genomes from 20 different taxa of the family *Leptotrichiaceae* aiming to provide the first such comparative analysis. Our study results confirm the picture of earlier phylogenies from this group that are now based on a larger scale of orthologous genes. We give a surveying insight into the investigated genomes, thereby also including recently described species from this family. With a novel approach it was, furthermore, possible to accurately and unequivocally type isolates of *S. moniliformis* based on three variable number tandem repeat (VNTR) sequences. With this, we are presenting a culture-independent, species-specific fingerprinting tool in order to type the most important causative organism of rat bite fever for the first time.

## Results

### Accession numbers

The GenBank/EMBL/DDBJ accession numbers for the genome sequences used in this study are summarized in Table 1.

**Table 1:** Strains as well as origins, clinical symptoms and host species of the *Leptotrichiaceae* members used in this study

Strain no.	Strain designation	Species	Year of isolation	Host	Clinic/sample	Country	Strain reference	Genome reference	Accession number
1	DSM 12112^T^ (=ATCC 14647^T^)	*Streptobacillus moniliformis*	1925	Human	Rat bite fever	France	[4]	[16]	CP001779.1CP001780.1
2	CIP 55-48	*Streptobacillus moniliformis*	1947	Mouse	Lymph adenitis	UK	n. d. a.	this study	LWQV00000000
3	ATCC 27747	*Streptobacillus moniliformis*	1964	Turkey	Septic arthritis	USA	[51]	this study	LWQW00000000
4	NCTC 10773	*Streptobacillus moniliformis*	1971	Human	Blood culture	UK	n. d. a.	this study	LYRU00000000
5	NCTC 11194	*Streptobacillus moniliformis*	1977	Human	Rat bite fever	UK	n. d. a.	this study	LWQX00000000
6	IPDH 144/80	*Streptobacillus moniliformis*	1980	Turkey	Septic arthritis	Germany	n. d. a.	this study	LWQY00000000
7	CIP 81-99	*Streptobacillus moniliformis*	1981	Human	Blood culture (wild rat bite)	France	n. d. a.	this study	LWSZ00000000
8	AHL 370-4	*Streptobacillus moniliformis*	1982	Mouse	Ear infection	Australia	n. d. a.	this study	LWTA00000000
9	NCTC 11941	*Streptobacillus moniliformis*	1983	Human	Haverhill fever	UK	n. d. a.	this study	LXKD00000000
10	IPDH 109/83	*Streptobacillus moniliformis*	1983	Turkey	Septic arthritis	Germany	n. d. a.	this study	LWTB00000000
11	ATCC 49567	*Streptobacillus moniliformis*	1989	Mouse	Lymph adenitis	Germany	[52]	this study	LWTC00000000
12	Kun 3 (RIVM)	*Streptobacillus moniliformis*	1991	Rat	Healthy	The Netherlands	[53]	this study	LWTD00000000
13	ATCC 49940	*Streptobacillus moniliformis*	1992	Rat	Otitis media	Germany	[54]	this study	LWTE00000000
14	B10/15	*Streptobacillus moniliformis*	Unknown	Wild rat	Unknown	The Netherlands	n. d. a.	this study	LWTF00000000
15	A378/1	*Streptobacillus moniliformis*	1995	Wild rat	Vaginal swab	Germany	DKFZ strain collection	this study	LWTG00000000
16	VA11257/2007	*Streptobacillus moniliformis*	2007	Human (farmer)	Rat bite fever, endocarditis	Germany	[55]	this study	LWTI00000000
17	VK105/14	*Streptobacillus moniliformis*	2008	Domestic rat	Abscess	Germany	TiHo strain collection	this study	LWTJ00000000
18	B5/1	*Streptobacillus moniliformis*	2009	Laboratory mouse	After rat bite	Germany	DKFZ strain collection	this study	LXKJ00000000
19	Marseille	*Streptobacillus moniliformis*	2009	Rat	Rat bite fever	La Réunion	[56]	this study	LXKI00000000
20	IKC1	*Streptobacillus moniliformis*	n. d. a.	Rat	Oral swab	Japan	[39]	this study	LXKH00000000
21	IKC5	*Streptobacillus moniliformis*	n. d. a.	Rat	Oral swab	Japan	[39]	this study	LXKG00000000
22	IKB1	*Streptobacillus moniliformis*	n. d. a.	Rat	Oral swab	Japan	[39]	this study	LXKF00000000
23	TSD4	*Streptobacillus moniliformis*	n. d. a.	Rat	Oral swab	Japan	[39]	this study	LXKE00000000
24	131000547^T^ (DSM 29248^T^)	*Streptobacillus felis*	2013	Cat	Pneumonia	Germany	[5, 7]	[18]	LOHX00000000
25	DSM 26322^T^ (HKU33^T^)	*Streptobacillus hongkongensis*	2014	Human	Abscess	Hong Kong	[8]	[18]	LOHY0000000
26	AHL 370-1^T^	*Streptobacillus notomytis*	1979	Spinifex hopping mouse	Sepicaemia, cultured from liver tissue	Australia	[57]	[6]	SAMN04038436
27	KWG2	*Streptobacillus notomytis*	n. d. a.	Rat (*Rattus rattus*)	Oral swab	Japan	[39]	this study	SAMN04099645
28	KWG24	*Streptobacillus notomytis*	n. d. a.	Rat (*Rattus rattus*)	Oral swab	Japan	[39]	this study	SAMN04099670
29	OGS16^T^	*Streptobacillus ratti*	n. d. a.	Rat (*Rattus rattus*)	Oral swab	Japan	[39]	[18]	SAMN04099675
30	CCUG 41628^T^	*Sneathia sanguinegens*	1999	Human	Blood	Sweden	[58, 59]	[38]	LOQF00000000
31	Sn35	*“Sneathia amnii”*	n. d. a.	Human	Vaginal microbiota	n. d. a.	[19]	[19]	NZ_CP011280
32	NCTC 11300^T^ (ATCC 33386^T^)	*Sebaldella termitidis*	1962	Termite	Intestine	n. d. a.	[60]	[17]	CP001739
33	DSM 1135 (C-1013-b)	*Leptotrichia buccalis*	2009	Human	Supragingival calculus	USA	n. d. a.	n. d. a.	CP001685
34	DSM 19756 (LB 57)	*Leptotrichia goodfellowii*	2013	Human	Prosthetic aortic valve	Germany	n. d. a.	n. d. a.	NZ_AZXW00000000
35	F0264	*Leptotrichia goodfellowii*	n. d. a.	Human	Oral cavity	n. d. a.	n. d. a.	n. d. a.	NZ_ADAD00000000
36	F0254	*Leptotrichia hofstadii*	n. d. a.	n. d. a.	n. d. a.	n. d. a.	n. d. a.	n. d. a.	NZ_ACVB00000000
37	DSM 19757	*Leptotrichia shahii*	2013	Human	Gingivitis	Norway	n. d. a.	n. d. a.	NZ_ARDD00000000
38	DSM 19758	*Leptotrichia wadei*	2004	Human	Saliva	Norway	[2]	n. d. a.	NZ_ARDS00000000
39	F0279	*Leptotrichia wadei*	n. d. a.	Human	Subgingival plaque	n. d. a.	n. d. a.	n. d. a.	NZ_AWVM00000000
40	Str. W10393	*Leptotrichia* sp. oral taxon 212	2015	Human	Oral microbiome project	n. d. a.	n. d. a.	n. d. a.	CP012410
41	Str. W9775	*Leptotrichia* sp. oral taxon 215	2015	Human	Oral microbiome project	n. d. a.	n. d. a.	n. d. a.	NZ_AWVR00000000
42	Str. F0581	*Leptotrichia* sp. oral taxon 225	2015	Human	Oral microbiome project	n. d. a.	n. d. a.	n. d. a.	NZ_AWVS00000000
43	Str. F0557	*Leptotrichia* sp. oral taxon 879	2015	Human	Oral microbiome project	n. d. a.	n. d. a.	n. d. a.	NZ_AWVL00000000
44	CCUG 39713^T^	*Caviibacter abscessus*	1998	Guinea pig	Cervical abscess	Sweden	n. d. a.	[38]	LOQG00000000
45	1510011837	*Caviibacter abscessus*	2015	Guinea pig	Cervical abscess	Germany	[38]	[38]	LOQH00000000
46	AVG2115^T^	*Oceanivirga salmonicida*	1992	Atlantic salmon	Septicaemia	Ireland	[32, 61]	[37]	LOQI00000000
47	ATCC 25586	*Fusobacterium nucleatum* subsp. *nucleatum*	n. d. a.	Human	Cervico-facial lesion	n. d. a.	n. d. a.	[62]	AE009951

^*T*^ type strain, *n. d. a.* no data available, *ATCC* American Type Culture Collection, Rockville, USA, *NCTC* National Collection of Type Cultures, London, UK, *CIP* Collection Institut Pasteur, Paris, France, *IPDH* Institute for Poultry Diseases, Hannover, Germany, *RIVM* Rijksinstituut voor Volksgezondheid en Milieuhygiene, Bilthoven, The Netherlands, *AHL* Animal Health Laboratory, South Perth, Australia, *ZfV* Zentralinstitut für Versuchstierzucht, Hannover, Germany, *DKFZ* Deutsches Krebsforschungszentrum, Heidelberg, Germany, *TiHo* Tierärztliche Hochschule Hannover, Germany, *RBF* rat bite fever

### Phylogenetic analysis based on orthologous genes

**Fig. 1:**
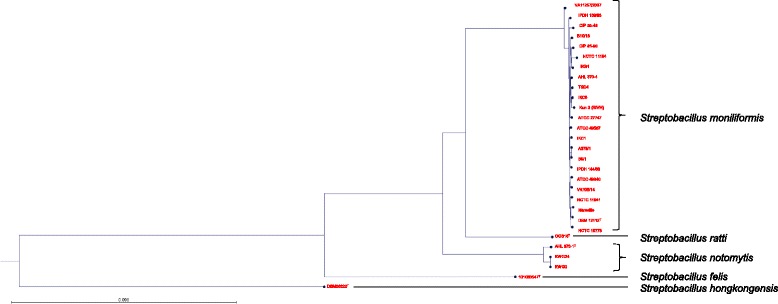
Maximum likelihood phylogenetic tree of the genus *Streptobacillus* (strains 1–29 according to Table 1). The tree is based on 281 orthologous genes including 57,841 SNPs

**Fig. 2:**
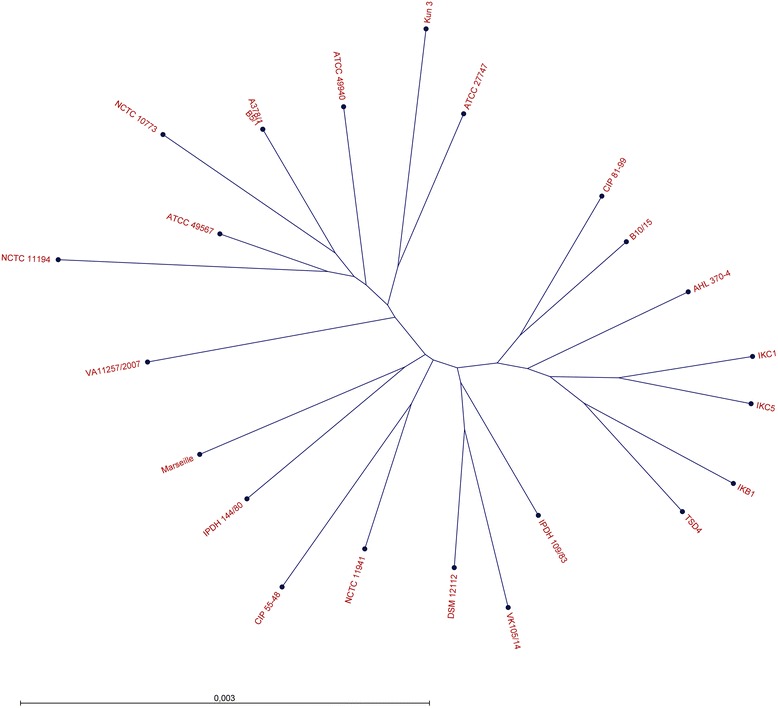
Unrooted maximum likelihood phylogenetic tree of 23 *Streptobacillus moniliformis* strains from this study. The tree is based on 775 orthologous genes including 5,211 SNPs

To determine the phylogeny within the genus *Streptobacillus* we aligned the allelic variations of 281 orthologous genes from 29 strains of *S. moniliformis*, *S. ratti*, *S. notomytis*, *S. felis* and *S. hongkongensis* which resulted in 57,841 single nucleotide polymorphisms (SNPs). From these SNPs we inferred a maximum likelihood phylogeny showing the distance between the different species within this genus (Fig. 1). To zoom deeper into the phylogeny of the *S. moniliformis* group we repeated this analyses with 775 orthologous genes present in 23 *S. moniliformis* strains which resulted in 5,211 SNPs. These SNPs were also used to construct a maximum likelihood phylogeny (Fig. 2).

As shown in the tree, most *S. moniliformis* strains used for this study are unrelated and form a heterogeneous population without any significant clustering. Solely strains A378/1 and B5/1 that both originate from the same source but without a common epidemiological background were phylogenetically indistinguishable.

### Analysis of genomes and protein functions

The genome size in members of the *Leptotrichiaceae* varies between 1.22 and 4.42 Mbp with *Caviibacter* (*C*.) *abscessus* and *Sebaldella* (*Se.*) *termitidis* being the smallest und largest genomes, respectively. Generally, and with the exception of *Sebaldella termitidis*, genomes are smaller than 2.45 Mbp. The genera *Caviibacter* and *Sneathia* (*Sn*.) are comparable with respect to genome size (1.22–1.34 Mbp) as are the genera *Streptobacillus* and *Oceanivirga* (*O*.) (1.38–1.90 Mbp). Members of the genus *Leptotrichia* (*L*.) are the second largest group with 2.31–2.47 Mbp. A general overview on the genomes of all strains under study is depicted in Table 2. A similar order can be observed with respect to coding DNA sequences (CDS), i.e., *C. abscessus* and *Sneathia* spp. possess 1212–1282 CDS, followed by *Streptobacillus* spp. and *O. salmonicida* (1293–1679), *Leptotrichia* spp. (1930–2365) and *Sebaldella termitidis* (4083). The average percentage of CDS within the whole genome displays a graded distribution within the family: a highly coding group consisting of the genera *Caviibacter, Oceanivirga* and *Sneathia* (89–93 %), an intermediate *Streptobacillus* spp. group (87 %) and a group containing the genera *Leptotrichia* and *Sebaldella* (84 %) with lower coding density. Nevertheless, intra-genus variability can be considerably high, the former results can inevitably also be shown for the average gene densities and the average intergenic regions (in parentheses average genes/Mbp; number of intergenic nt): *O. salmonicida* (1056; 79), *C. abscessus* (996; 76), *Sneathia* spp. (989; 84), *Streptobacillus* spp. (987; 115), *Leptotrichia* spp. (967; 144) and *Sebaldella* (936; 149). An organization of the genomes under study into clusters of orthologous groups (COGs) is depicted in Additional files 1 and 2 and shows, however, high intra-species as well as inter-species variations. On a generic level, gene contents of COG classes J, L, D and F are inversely correlated with increasing genome size, whereas COG classes K, N, T and Q are positively correlated (see Additional files 1 and 2).

**Table 2:** Analysis of genome data as well as predictions of coding regions of the *Leptotrichiaceae* members used in this study

Strain no.	Organism	Approx. genome size (nt)	CDS^a^	rRNA	tRNA^b^	% GC^c^	Total DNA coding regions (nt)	Total non-coding regions (nt)	Coding genome space (%)	Average gene density (genes/Mbp)	Average inter-genic region (nt)
1	*S. moniliformis*	1673280	1568	16	39	26.3	1556870	116410	93	937	74
2	*S. moniliformis*	1678906	1658	12	37	26.1	1508835	170071	89	988	103
3	*S. moniliformis*	1684459	1591	14	35	26.1	1486041	198418	87	945	125
4	*S. moniliformis*	1897024	2244	9	43	28.9	1651665	245359	85	1183	109
5	*S. moniliformis*	1712153	1764	3	38	26.1	1542831	169322	89	1030	96
6	*S. moniliformis*	1668382	1615	13	36	26.1	1484745	183637	88	968	114
7	*S. moniliformis*	1686977	1543	12	35	26.4	1449924	237053	84	915	154
8	*S. moniliformis*	1598404	1608	14	38	25.9	1470174	128230	91	1006	80
9	*S. moniliformis*	1689124	1675	4	36	26.1	1399686	289438	79	992	173
10	*S. moniliformis*	1756513	1765	14	37	26.1	1559103	197410	87	1005	112
11	*S. moniliformis*	1763717	1621	9	35	26.1	1488168	275549	81	919	170
12	*S. moniliformis*	1518628	1540	12	33	25.9	1442043	76585	95	1014	50
13	*S. moniliformis*	1689360	1765	5	36	26.1	1526748	162612	89	1045	92
14	*S. moniliformis*	1674237	1597	13	37	26.2	1477515	196722	87	954	123
15	*S. moniliformis*	1667701	1692	14	36	26.0	1518810	148891	90	1015	88
16	*S. moniliformis*	1690579	1538	16	37	26.1	1468143	222436	85	910	145
17	*S. moniliformis*	1608659	1507	22	34	26.2	1433763	174896	88	937	116
18	*S. moniliformis*	1497161	1644	8	36	25.8	1322022	175139	87	1098	107
19	*S. moniliformis*	1696954	1774	5	38	26.1	1521612	175342	88	1045	99
20	*S. moniliformis*	1696554	1688	17	37	26.0	1509528	187026	88	995	111
21	*S. moniliformis*	1792325	1664	16	42	26.2	1550631	241694	84	928	145
22	*S. moniliformis*	1759287	1737	13	43	25.9	1566621	192666	88	987	111
23	*S. moniliformis*	1608076	1559	10	35	26.0	1445580	162496	89	969	104
24	*S. felis*	1610666	1754	3	37	26.4	1450014	160652	89	1089	92
25	*S. hongkongensis*	1543001	1485	14	35	26.1	1324059	218942	83	962	147
26	*S. notomytis*	1762984	1773	9	43	28.1	1511157	251827	83	1006	142
27	*S. notomytis*	1426245	1349	8	40	26.4	1257996	168249	87	946	125
28	*S. notomytis*	1384502	1341	19	39	26.3	1256817	127685	90	969	95
29	*S. ratti*	1499353	1411	11	39	25.9	1318767	180586	86	941	128
30	*Sneathia sanguinegens*	1300753	1329	2	34	26.7	1214541	86212	93	1022	65
31	*“Sn. amnii”*	1339284	1282		34	28.3	1207722	131562	89	957	103
32	*Sebaldella termitidis*	4418842	4135	13	40	33.5	3802074	616768	84	936	149
33	*Leptotrichia buccalis*	2465610	2299	15	46	29.6	2062809	402801	80	932	175
34	*L. goodfellowii*	2281162	2241	7	39	31.6	2045213	235949	88	982	105
35	*L. goodfellowii*	2287284	2373	3	39	31.5	2055020	232264	89	1037	98
36	*L. hofstadii*	2453253	2720	13	47	30.8	2059248	394005	81	1109	145
37	*L. shahii*	2144606	1969	10	41	29.5	1812950	331656	82	918	168
38	*L. wadei*	2316529	2139	11	42	29.3	1973929	342600	83	923	160
39	*L. wadei*	2353455	2212	3	27	29.2	2008568	344887	83	940	156
40	*Leptotrichia* sp. oral taxon 212	2444904	2231	14	43	31.4	2146482	298422	86	936	130
41	*Leptotrichia* sp. oral taxon 215	2308492	2195	3	34	31.4	2039067	269425	87	951	123
42	*Leptotrichia* sp. oral taxon 225	2400083	2306	3	24	29.6	2061283	338800	84	961	147
43	*Leptotrichia* sp. oral taxon 879	2415750	2361	4	25	29.6	2026284	389466	81	977	165
44	*C. abscessus*	1219935	1198			26.5	1131456	88479	92	982	74
45	*C. abscessus*	1304155	1316	4	35	26.4	1201320	102835	91	1009	78
46	*O. salmonicida*	1769081	1869	2	38	25.4	1621182	147899	91	1056	79
47	*Fusobacterium nucleatum* (outgroup)	2174500	2022	15	47	27.2	1937724	236776	88	930	117

^a^CDS: DNA coding sequences; ^b^tRNA: transfer ribonucleic acid; ^c^GC: guanine-cytosine content

### Multiple-Locus Variable number tandem repeat Analysis (MLVA)

#### *In silico* VNTR analysis

Under default conditions, 127 repeats were identified by the tandem repeat finder. For further analysis, the three most variable VNTRs were identified according to the degree of variability of allele types identified by alignment analysis (Table 3). These three allelic loci were only present in *S. moniliformis* and thus proved to be specific for this microorganism (all other members of the *Leptotrichiaceae* were negative). The combination of the three loci yielded a high discriminatory index (0.94296 DI; Table 4).

**Table 3:** *Streptobacillus moniliformis* specific Variable Number of Tandem Repeat (VNTR) primer sequences used in this study

Primer ID	VNTR position^a^	Repeat size in nt (identity in %)	Sequence (5–3)	PCR product size (bp)
VNTR_Sm1	1576120 - 1576145	3 (100)	TCA TTT ACT CAC CCT AGT AGT GGT	210
			CCA GTT GAA TAT AAG CTT GCT ATG G	
VNTR_Sm2	1182890 - 1182907	6 (100)	TGG AAC TGT TTG TTG AGT ATT TCC A	298
			AGG GAC AGA TGT TCA ATT TGT GTA	
VNTR_Sm3	284997 - 285268	36 (91)	TAC GCT GTA GGG TTG AAC GG	830
			ACA GTT TGA GCA CGT CTT AAT CC	

Primers were designed with Geneious (v. 8.1.3; Biomatters, Auckland, NZ) [43] and to be complementary to VNTR flanking regions that were conserved among genomes; ^a^according to the *S. moniliformis* DSM 12112^T^ genome (CP001779.1)

**Table 4:** VNTR allele types of the *Streptobacillus moniliformis* strains used in this study

Isolate ID	VNTR_Sm1^a^	VNTR_Sm2	VNTR_Sm3^b^	Allele code
**DSM 12112** ^**T**^	**9**	**3**	**16**	**LHL1**
**CIP 55-48**	**7**	**3**	**16**	**LHL10**
**ATCC 27747**	**10**	**3**	**16**	**LHL4**
NCTC 10773	8	4	17	LHL15
NCTC 11194	6	3	17	LHL16
IPDH 144/80	6	3	16	LHL5
CIP 81-99	7	3	16	LHL10
AHL 370-4	7	2	15	LHL3
NCTC 11941	6	3	18	LHL11
IPDH 109/83	6	3	16	LHL5
ATCC 49567	6	3	16	LHL5
**Kun 3 (RIVM)**	**6**	**3**	**18**	**LHL11**
ATCC 49940	6	3	14	LHL6
**B10/15**	**6**	**4**	**15**	**LHL7**
**A378/1**	**8**	**5**	**16**	**LHL2**
VA11257/2007	6	3	16	LHL5
VK105/14	8	3	16	LHL13
B5/1	8	5	16	LHL2
Marseille	6	4	14	LHL14
IKC1	6	3	15	LHL8
**IKC5**	**5**	**3**	**15**	**LHL9**
**IKB1**	**6**	**3**	**16**	**LHL5**
**TSD4**	**11**	**3**	**18**	**LHL12**
**A40-13** ^**c**^	**11**	**2**	**17**	**LHL17**

Bold rows represent strains used for a PCR-based validation of *in silico* identified VNTR allele types (underlined alleles were not found *in silico* and only identified after PCR amplification); ^a^ in order to fit requirements of the database, the repeat copy numbers at locus VNTR_Sm1 have been rounded up to receive integer values (e.g., 9 instead of 8.7); ^b^while the repeat copy numbers at locus VNTR_Sm3 have been rounded up to the next half-value and doubled to receive integer values (e.g., 15 instead of 7.2); ^T^: type strain; ^c^strain was only used for validation (no complete genome available)

### PCR-based validation of *in silico* results

The absence of the calculated VNTR loci could also be proven by polymerase chain reaction (PCR) in all *Leptotrichiaceae* members other than *S. moniliformis* (data not shown). Contrarily, each of the ten *S. moniliformis* strains exhibited a specific band corresponding to their predicted tandem repeats pattern. Analysis of the sequenced PCR products confirmed the allele type allocation determined *in silico* (Table 4). VNTR_Sm1 alleles of two isolates, which were not found *in silico*, were successfully assigned (Table 4). Re-calculation revealed a DI of 0.9529 after including these two isolates, as well as one isolate for which no genome data was available. In order to facilitate comparisons of results in future studies, every genotype (from the allele types of the three loci) was expressed as a specific allele combination resulting in a specific allele code (Table 4). An online database dedicated to MLVA results of *S. moniliformis* has been established on the webserver of University Paris-Saclay, Orsay, France (http://microbesgenotyping.i2bc.paris-saclay.fr/databases/public) which is open to future entries and strain comparisons.

## Discussion

Members of the *Leptotrichiaceae* are rarely encountered microorganisms, a phenomenon that seems to be highly dependent on difficulties with cultivation. With the availability of molecular methods in this field the number of findings and frequencies has significantly increased [10–15, 32–36]. On the other hand, we still need deeper insight into the genomes of this group. In particular, the mechanisms involved in pathogenesis and virulence of pathogenic species are completely unexplored. We have undertaken a first step into this direction by analysing a broad spatio-temporal collection of strains, thereby including especially species with regular evidence for pathologies. Firstly, the large dataset from this study has been utilized for the confirmation of our phylogenetic picture from earlier studies [18, 30, 37, 38]. An intra-genus phylogeny that was based on 775 orthologous genes revealed a very similar picture to previous studies involving only four selected functional genes (Figs. 1 and 2). Conversely and in contrast to almost identical average nucleotide identity (ANI) values [30], full genome analyses revealed a high level of heterogeneity for all but two strains (no. 15 and 18) of *S. moniliformis* without any significant clustering. This is, albeit, not surprising, because the present study included a large spatio-temporal collection of 23 *S. moniliformis* strains that have been isolated over a period of 90 years from at least five different host species and from almost all subcontinents. We were also able to display the three predicted *Leptotrichiaceae* specific CSIs of MreB/MrI (2 aa deletion), AlaS and RecA (5 and 2 aa insertions, respectively) in all of our genomes as well as in the recently described members of the family (data not shown) [31].

Genome size dependent gene content has been described and could also be confirmed for the genomes from this study [19]. With increasing genome size gene contents of COG classes J, L, D and F involved in DNA replication, cell cycle regulation and protein translation are inversely correlated, whereas COG classes K, N, T and Q involved in transcription, signal transduction, cell motility and the biochemistry of secondary metabolites are positively correlated (see Additional files 1 and 2). This makes sense when essential gene functions are preserved in smaller genomes and less important gene functions which are dispensable or can be ‘outsourced’ to the host, are lost [19]. On first impression the group of *S. moniliformis* strains is highly similar as can be concluded from related morphological and phenotypical properties and also from their high intra-species ANI of 98.5–99.3 % (cf. Table S2 in [30]). Based on data from this study very similar COG classes were also observed within this group (see Additional files 1 and 2), but differences in coding densities suggested, on the other hand, remarkable discrepancies. Fuelled by the idea that these discrepancies could, furthermore, be utilized with respect to epidemiology, we have developed a specific MLVA typing scheme for the major pathogen from this group, *S. moniliformis*, and the causative organism of rat bite fever. This scheme proved to be sufficient in unequivocally typing all 23 *S. moniliformis* strains under study plus one additional isolate with high discriminatory power (0.9529 DI). Interestingly, only four allele codes (genotypes; LHL2, LHL5, LHL10 and LHL11) were found more than once among isolates (Table 4). At least for LHL2 isolates, a connection could be pursued in that both isolates have been stored in the same strain collection, although a direct transmission could not be proven. To check the clonality of isolates belonging to these four genotypes we have investigated further loci with high discriminatory potential, i.e., the clustered regularly interspaced short palindromic repeats (CRISPR) region known to occur in *S. moniliformis* (http://crispr.u-psud.fr/cgi-bin/crispr/SpecieProperties.cgi?Taxon_id=519441). In contrast to all other allele codes (LHL5, LHL10, LHL11), both strains (no. 15 and 18) belonging to the allele code LHL2 indeed shared an identical CRISPR region, thereby pointing towards a clonal relation of these two isolates (data not shown) as could also be concluded from the phylogenetic tree (Fig. 2). Due to its length of up to approximately 3,000 nucleotides and its high level of heterogeneity the CRISPR region seems, on the other hand, presently not very well suited as a direct typing tool, but could be useful in certain situations to confirm or negate clonality of strains. A second advantage of the MLVA method described in this study is that it can effectively be pursued directly from the original matrix (e.g., a mouth microbiota swab and a clinical sample) without prior cultivation of the organism, which offers the possibility to better understand transmission chains in the future. This seems to be especially relevant since established PCR assays are not species specific, but limited to genus level specificity [37, 39, 40]. The majority of diagnoses of rat bite fever cases in the recently published literature relies only on partial 16S rRNA gene sequence analysis that may – in the light of very similar novel *Streptobacillus* spp. that also colonize rats – be quite uncertain for proper pathogen identification [41]. Hopefully, the newly established MLVA database will help to clarify regional infectious clusters and confirm transmission of certain lineages.

## Conclusion

We have undertaken a first analysis of *Leptotrichiaceae* genomes using a large spatiotemporal collection of strains also including novel members of this group. Our dataset unveiled a first insight into characteristics founding a stable phylogeny, genome structure and COG classes. Beside apparent intra-species similarities we have detected also genetic heterogeneities that provided a basis for fingerprinting the most relevant pathogen from this group, the rat bite fever organism, *S. moniliformis*. This highly useful and economical tool can be directly used from clinical samples without ambitious prior cultivation and with high discriminatory power. Our data form the basis for a newly established MLVA database that provides the opportunity to store and compare isolate-specific information in future cases with this neglected zoonosis.

## Methods

### Generation of genomic data

Twenty-two strains of *S. moniliformis* were sequenced in this study, ten strains were taken from previous publications of our group and 15 strains were descended from other projects (Table 1). Genomic DNA was extracted from a 72 h bacterial culture with a commercial kit according to the manufacturer’s instructions (MasterPure™ Complete DNA and RNA Purification Kit, Epicentre, distributed by Biozym Scientific, Hessisch Oldendorf, Germany). Whole genome sequencing of the strains was performed on an Illumina MiSeq with v3 chemistry resulting in 300 bp paired end reads and a coverage of greater than 90×. Quality trimming and de novo assembly was performed with CLC Genomics Workbench, Version 7.5 (CLC Bio, Aarhus, Denmark). For automatic annotation we used the RAST Server: Rapid Annotations using Subsystems Technology [42]. Data from further relevant reference genomes from the *Leptotrichiaceae* were also utilized and obtained from the National Center for Biotechnology Information (NCBI) database (http://www.ncbi.nlm.nih.gov). Sequence analyses and genome calculations as well as oligonucleotide primer generation were carried out with Geneious (v. 8.1.3; Biomatters, Auckland, NZ) [43]. Table 1 depicts the set of strains and reference genomes used for this study.

### Phylogenetic analysis based on orthologous genes

The determination of the maximum common genome (MCG) alignment was done comprising those genes present in all genomes considered for comparison [44]. Based on the parameters sequence similarity (minimum 70 %) and coverage (minimum 90 %) the genes were clustered and those genes that were present in each genome, fulfilling the threshold parameters were defined as MCG. This resulted in 281 orthologous genes for the comparison of 29 strains of *S. moniliformis, S. ratti, S. notomytis, S. felis* and *S. hongkongensis* and in 775 orthologous genes for the comparison within 23 strains of *S. moniliformis* only.

The following extraction of the allelic variants of these genes from all genomes was performed by a blast based approach after which they were aligned individually for each gene and concatenated which resulted in an alignment of 219,961 bp for the 29 strains and of 546,508 bp for the 23 *S. moniliformis* strains [45].

This alignment was used to generate a phylogenetic tree with randomized axelerated maximum likelihood (RAxML) 8.1 [46] using a General Time Reversible model and gamma correction for among site rate variation.

### Analysis of genomes and protein functions

Genes were predicted with Prodigal [47] and assigned to COGs with the NCBI’s Conserved Domain Database [48].

### Multiple-Locus Variable number tandem repeat Analysis (MLVA)

#### *In silico* VNTR analysis

The complete genome sequence of the *S. moniliformis* type strain DSM12112^T^ (accession number CP001779.1) was used to search for potential VNTRs using a tandem repeat finder web tool (http://tandem.bu.edu/trf/trf.basic.submit.html). We focused our search on repeats that were characterized by high purity, large size, and/or large number of repeat copies [49]. Repeats of interest were aligned against a set of available genomes depicted in Table 1 using Geneious and allele types were determined as shown in repeat copy numbers. The DI was calculated for a combination of three most variable VNTRs using an online discriminatory power calculator (http://insilico.ehu.es/mini_tools/discriminatory_power/).

### PCR-based validation of *in silico* results

Ten *S. moniliformis* strains (strain nos. 1, 2, 3, 12, 14, 15, 21, 22 and 23 according to Table 1 plus strain A40-13 for which complete genomic data were not available) as well as all accessible members of the *Leptotrichiaceae* other than *S. moniliformis* were used for validation. DNA was extracted from respective isolates (2–3 colonies) by boiling in 100 μL distilled water for 20 min (min.) followed by centrifugation at 20,817 × *g* for 5 min. The 20 μL final PCR reaction contained 10 μL of Hotstar Taq MasterMix (Qiagen, Hilden, Germany), 1 μL of each forward and reverse primer (10 pmol/μL) (TIB MOLBIOL, Berlin, Germany) (Table 3), 6 μL DNase free PCR grade water (Qiagen), and 2 μL of the extracted DNA. PCR conditions were as following: 1× (95 °C, 15 min), 40x (94 °C, 30 s; 58 °C, 30 s; 72 °C, 30 s), 1× (72 °C, 10 min). PCR products were stained with ethidium bromide in a 2 % agarose gel (100 V for 1.5 h) and then analyzed with a gel documentation system (BioDoc-It, UVP, UK). The PCR amplicons were purified using MicroElute DNA Cycle-Pure Kit (OMEGA bio-tek, Norcross, USA) and sequenced at Seqlab-Microsynth laboratories (Göttingen, Germany). All sequences were analyzed by tandem repeat finder web tool and/or BLASTN 2.3.1+ [50] hosted by NCBI website and compared to the *in silico* results.

## Additional files

Additional file 1: Table S1. Analysis of clusters of orthologous groups (COGs) of the *Leptotrichiaceae* members used in this study. COGs were assessed as described in the Materials and Methods. (XLSX 16 kb)
Additional file 2: Figure S1. Relative abundances of clusters of orthologous groups (COGs) of the *Leptotrichiaceae* members used in this study. COGs were assessed as described in the Materials and Methods. (TIF 4188 kb)

## Abbreviations

AHL: Animal Health Laboratory South Perth, Australia
ANI: Average nucleotide identity
ATCC: American Type Culture Collection Rockville, USA
*C*.: *Caviibacter*
°C: Degrees Celsius
CDS: Coding DNA sequences
CIP: Collection Institut Pasteur Paris, France
COG: Cluster of orthologous groups
CRISPR: Clustered regularly interspaced short palindromic repeat
CSI: Conserved signature indel
DDBJ: DNA Data Bank of Japan
DI: Discriminatory index
DNA: Desoxyribonucleic acid
DKFZ: Deutsches Krebsforschungszentrum Heidelberg, Germany
EMBL: European Molecular Biology Laboratory
Fig.: Figure
*g*: Gravity
GC: Guanine-cytosine content
h: Hour
IPDH: Institute for Poultry Diseases Hannover, Germany
kDa: kilo Dalton
*L.*: *Leptotrichia*
LHL: Landesbetrieb Hessisches Landeslabor
Mbp: Mega base pairs
MCG: Maximum common genome
min: minute
MVLA: Multi locus variable number tandem repeat analysis
μL: micro liter
NCBI: National Center for Biotechnology Information
NCTC: National Collection of Type Cultures London, UK
n. d. a.: no data available
nt: nucleotides
*O*.: *Oceanivirga*
PCR: Polymerase chain reaction
RAST: Rapid annotations using subsystems technology
RaxML: Randomized axelerated maximum likelihood
RBF: Rat bite fever
RIVM: Rijksinstituut voor Volksgezondheid en Milieuhygiene Bilthoven, The Netherlands
rRNA: ribosomal ribonucleic acid
*S.*: *Streptobacillus*
*Se.*: *Sebaldella*
sec.: second
*Sn.*: *Sneathia*
SNPs: Single nucleotide polymorphisms
^T^: Type strain
TiHo: Tierärztliche Hochschule Hannover, Germany
tRNA: transfer ribonucleic acid
VNTR: Variable number tandem repeat
ZfV: Zentralinstitut für Versuchstierzucht, Hannover, Germany
