# Supplementary figures and images for: Phylogenetic and comparative genomics of the family Leptotrichiaceae and introduction of a novel fingerprinting MLVA for Streptobacillus moniliformis
Source: BMC Genomics. 2016 Nov 3;17:864. doi: 10.1186/s12864-016-3206-0 (PMC5093955; doi:10.1186/s12864-016-3206-0)

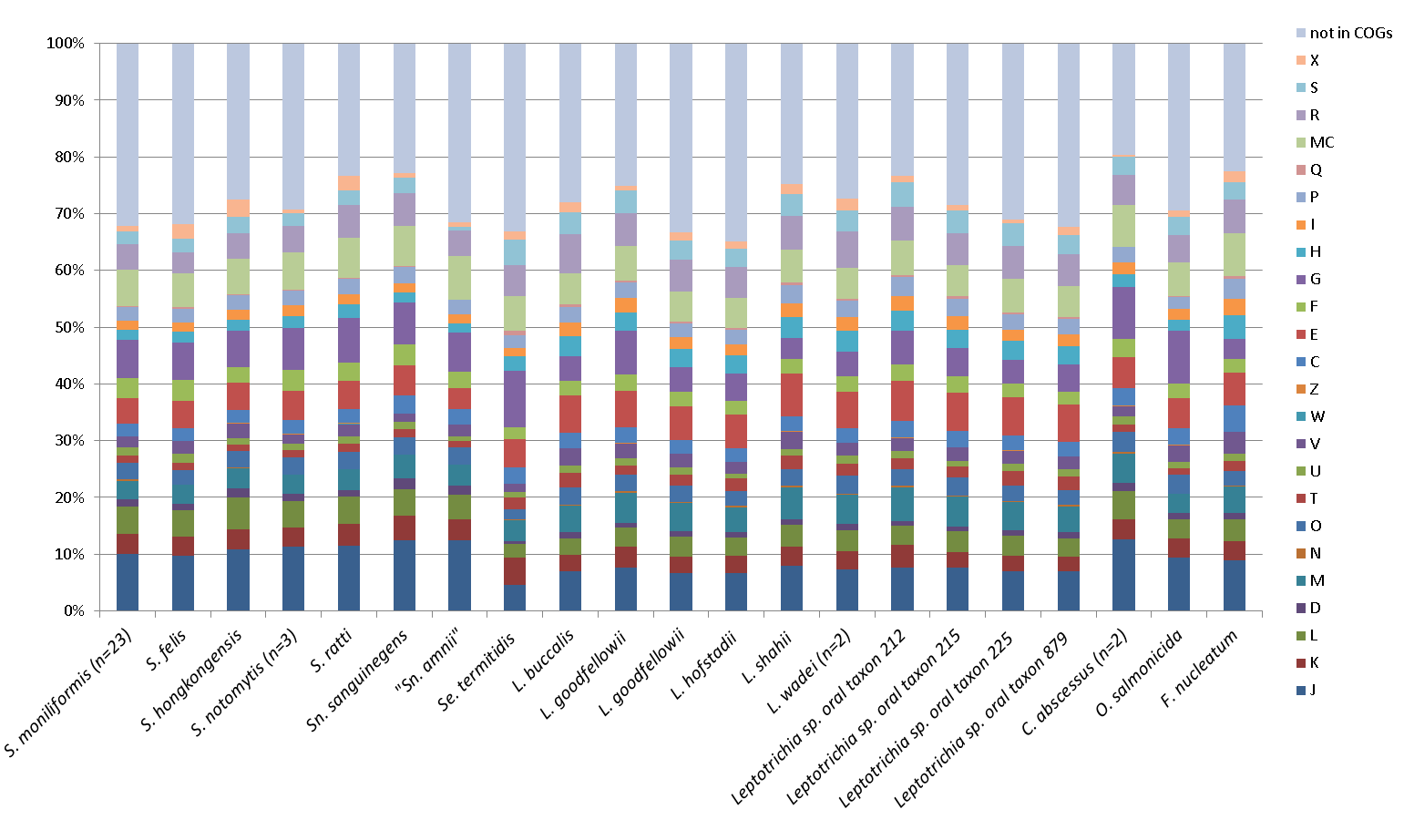

Supplement: Additional file 2: Figure S1. — Relative abundances of clusters of orthologous groups (COGs) of the Leptotrichiaceae members used in this study. COGs were assessed as described in the Materials and Methods. (TIF 4188 kb) [file 12864_2016_3206_MOESM2_ESM.tif]
